# Randomized Phase IIA Trial of Gemcitabine Compared With Bleomycin Plus Vincristine for Treatment of Kaposi’s Sarcoma in Patients on Combination Antiretroviral Therapy in Western Kenya

**DOI:** 10.1200/JGO.17.00077

**Published:** 2018-01-11

**Authors:** Naftali W. Busakhala, Paul J. Waako, Matthew Robert Strother, Alfred Kipyegon Keter, Gabriel Kimutai Kigen, Fredrick Chite Asirwa, Patrick J. Loehrer

**Affiliations:** **Naftali W. Busakhala**, **Gabriel Kimutai Kigen**, Moi University School of Medicine; **Alfred Kipyegon Keter**, and **Patrick J. Loehrer Sr**, AMPATH Statistics, Eldoret, Kenya; **Paul J. Waako**, Makerere College of Health Sciences, Kampala, Uganda; **Matthew Robert Strother**, Canterbury District Health Board, and University of Otago, Christchurch, New Zealand; **Fredrick Chite Asirwa**, Indiana University Simon Cancer Center, Indianapolis, IN.

## Abstract

**Purpose:**

Kaposi’s sarcoma (KS) is a spindle cell tumor resulting from growth dysregulation in the setting of infection with human herpes virus-8 (also called KS herpes virus). Advanced KS is characterized by poor responses to antiretroviral therapy and some of the chemotherapy readily accessible to patients in low-resource areas. Gemcitabine induced partial and complete regression of AIDS-associated KS (AIDS-KS) in 11 of 24 patients in a pilot study. The current study compares the antimetabolite gemcitabine with the standard care bleomycin and vincristine (BV) in the treatment of chemotherapy-naïve patients with AIDS-KS in a resource-limited setting.

**Patients and Methods:**

Patients with persistent or progressive KS despite treatment with combined antiretroviral therapy were randomly assigned to receive gemcitabine 1,000 mg/m^2^ or bleomycin 15 IU/ m^2^ and vincristine 1.4 mg/m^2^ given twice weekly. The main end point was objective response by bidirectional measurement, adverse events, and quality of life after three cycles of chemotherapy.

**Results:**

Of 70 participants enrolled, 36 received gemcitabine and 34 received BV. Complete response was achieved in 12 patients (33.3%) in the gemcitabine arm and six (17.6%) in the BV arm (*P* = .175). The partial response rate was 52.8% (n = 19) in the gemcitabine arm and 58.8% (n = 20) in the BV arm. Both study arms reported similar neurologic and hematologic adverse events; there was statistically significant baseline to post-treatment improvement in health-related quality-of-life scores.

**Conclusion:**

The results of this randomized, phase IIA trial demonstrate gemcitabine activity in chemotherapy-naïve patients with AIDS-KS, on the basis of response rates, adverse events, and health-related quality-of-life scores.

## INTRODUCTION

Kaposi’s sarcoma (KS) is a tumor composed of spindle cells of uncertain etiology and is caused by infection with human herpes virus-8. The incidence of KS among patients with AIDS is 20 times that of the general population, and KS is diagnosed in 15% of people infected with HIV who are not receiving combined antiretroviral therapy (cART).^1^ The high prevalence of HIV and human herpes virus-8 in Africa has led to an increased incidence of KS in sub-Saharan Africa.^2,3^ The primary treatment of AIDS-KS is cART: Up to 80% of patients can exhibit regression with immune reconstitution on cART in early KS.^4^ Patients with advanced disease rarely respond to cART alone and require concomitant chemotherapy. A trial from South Africa in patients with advanced KS compared cART with cART plus chemotherapy and reported a response rate of 39% with cART alone compared with 66% in the cART plus chemotherapy arm, with 35% crossover from the cART-only arm.^5^

Several chemotherapeutic agents have undergone clinical trials for treatment of KS, including doxorubicin, paclitaxel, bleomycin, vincristine, and vinblastine. Low responses or high toxicity were reported for interferon-α, etoposide, thalidomide, lenalidomide, sirolimus, and bevacizumab, and these are not recommended.^6-9^ Liposomal doxorubicin is the standard chemotherapy for KS in developed countries; but, in many resource-limited regions, patients are treated with the relatively inexpensive and available bleomycin (15 mg/m^2^) plus vincristine (1.4 mg/m^2^) administered every 2 weeks for three to six cycles^10,11^ or with single-agent vincristine.^12,13^ Complete response rates vary from 25% to 30%, thus leaving > 70% of patients in need of alternative treatments.

The oncology clinic at Moi Teaching and Referral Hospital (MTRH) routinely uses gemcitabine for treatment of several malignancies.^14-19^ Supplies of gemcitabine donated by Eli Lilly (Indianapolis, IN) afford the continuity of treatment even in this poorly insured population. In addtion, drugs such as gemcitabine or nonvesicant oral agents are preferable in low- and middle-income countries because of a shortage of skilled oncology nurses to deliver vesicant chemotherapeutic agents.

Because of its reported activity in sarcomas and lack of cross-resistance with bleomycin plus vincristine (BV) therapy, gemcitabine was retrospectively evaluated in patients with KS treated through Academic Model Providing Access to Healthcare (AMPATH).^20^ Gemcitabine administered at a dose of 1,000 mg every 2 to 4 weeks was used at MTRH as a second-line medication in a pilot study of 24 patients in 2010. This alternative schedule to the weekly gemcitabine was selected because of transportation issues and concerns regarding adequate supportive care for myelosuppression. Objective response, as determined by serial skin examinations, was found in 11 patients (complete response [CR], n = 3; partial response [PR], n = 8; CR and PR = 48%); 11 were classified with stable disease and one with progressive disease. Clinical benefit response, as determined by decreases in pain medication use, weight gain, and improvement in Karnofsky Performance Status, was observed in 15 patients (65%). The progression-free survival was 0 to 3 months. After follow-up for 2 to 10 months, two patients were still receiving chemotherapy and three were lost to follow-up. Eighteen patients completed chemotherapy, five progressed, and one died of a secondary malignancy, hepatoma.^20^ Given these compelling preliminary data, and with the intention to increase chemotherapeutic options for AIDS-KS, the AMPATH Oncology Institute initiated in 2013 a phase IIA trial of gemcitabine as first-line chemotherapy.

## PATIENTS AND METHODS

### Patients

Inclusion criteria were age ≥ 18 years, histology-confirmed KS, and serologically confirmed HIV infection. KS histology was performed by local pathologists and all slides were sent to University of California, San Francisco, for confirmation by dermatopathologists.^21^ All subjects had to be receiving first-line cART composed of two nucleoside and one nonnucleoside reverse transcriptase inhibitor for at least 8 weeks, although all participants had been receiving cART for > 3 months. This meant none of the participants with progressive disease had immune reconstitution syndrome. Additional inclusion criteria were adequate end-organ function, Eastern Cooperative Oncology Group performance status score ≤ 2, and a negative pregnancy for female participants. Exclusion criteria included previous chemotherapy; allergy to gemcitabine, bleomycin, or vincristine; difficult-to-measure lesions; concurrent malnutrition; malignancies or infections including active tuberculosis and pregnancy or breastfeeding for female participants. All participants signed a voluntary informed consent and the protocol was approved by the Institutional Ethics and Research Committee of Moi University/MTRH and the Ministry of Health’s Expert Committee on Clinical Trials.

### Study Design

Patients were randomly assigned 1:1 to receive either gemcitabine or BV. Those randomly assigned to the gemcitabine arm received gemcitabine 1,000 mg/m^2^ every 2 weeks, whereas those randomly assigned to the BV arm received bleomycin 15 IU/m^2^ plus vincristine 1.4 mg/m^2^ every 2 weeks. The maximum dose of vincristine was 2 mg per cycle.

Disease assessment was performed by visual inspection. Cutaneous KS lesions were counted, documented, and photographed. The most convenient lesions to access and measure were selected as the index lesions and measured in two directions using a Vernier caliper every 2 weeks. To avoid interobserver errors, only one study staff member measured and photographed the KS lesions.

Response was determined by clinical observation after three cycles of chemotherapy. This was because chemotherapy in AIDS-KS is adjuvant and if there is disease progression after two to three cycles, both chemotherapy and cART should be re-evaluated. Continuing with the same drugs is unlikely to change the response but may cause more toxicity. Participants transitioned to routine care and continued with observation if they had CR. Those with PR continued with gemcitabine or BV for six cycles, whereas those with stable or progressive disease were transitioned to appropriate standard care. Follow-up after three cycles for progression-free survival and overall survival was not done, because of the phase 11A study design.

Baseline laboratory values recorded were CD4 levels, CBC count, and renal and hepatic function, the latter of which were repeated before each cycle. Toxicity was assessed by the Common Terminology Criteria for Adverse Events version 4.^22^ Health-related quality of life (QoL) was assessed using the Functional Assessment of Cancer Therapy-Kaposi Sarcoma (FACT-KS) version 4 questionnaire.^23^ Blood transfusions and use of iron and folate were permitted per local standard care.

### Sample Size

This was a phase IIA (ie, proof of concept) clinical trial. We determined a sample size such that with 95% confidence and 80% certainty we would be able to detect a ≥ 20% improvement in the positive outcome among those in the gemcitabine arm.^24^ Therefore, we needed 35 patients per arm.

### Statistical Data Analysis

Data analysis was performed in R (https://www.r-project.org/). Categorical variables were summarized as frequencies and the corresponding percentages; continuous variables were summarized as mean and the corresponding standard deviation. Association between the exposure and other categorical variables was assessed using the Pearson χ^2^ test, and Fisher exact *P* values were reported when the χ^2^ assumptions were violated. Association between continuous variables and the exposure was assessed using Mann-Whitney U-test.

## RESULTS

### Characteristics of Study Participants

**Fig 1:**
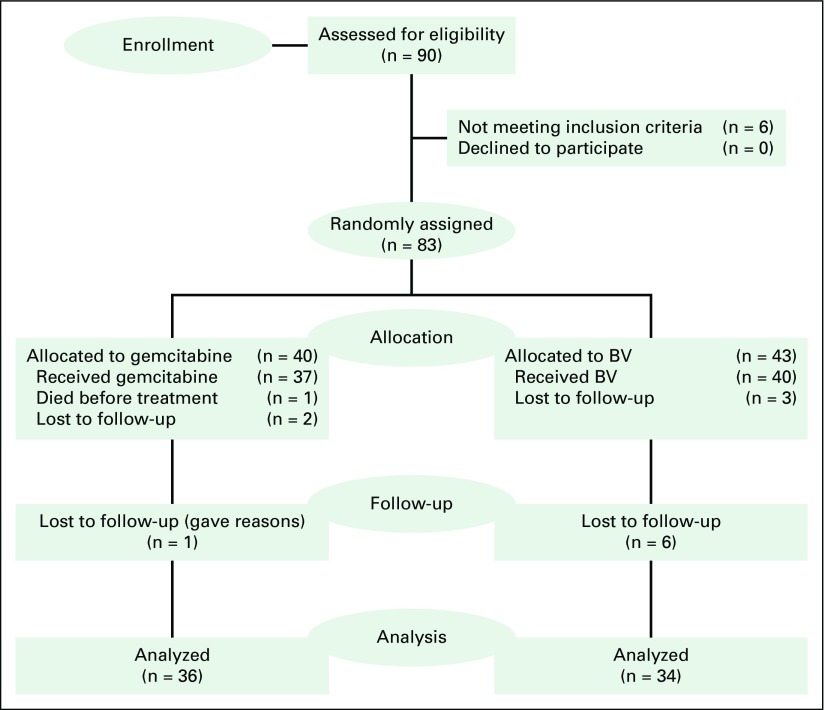
CONSORT diagram. Some study participants (n = 6) were lost to follow-up.

Between March 2013 and December 2015, 70 patients were recruited: 36 were randomly assigned to the gemcitabine arm and 34 to the BV arm (Fig 1). Patients in both arms of the study were comparable (Table 1). Male patients constituted 56% of the study cohort. Participants were between 19 and 70 years, with a median age of 35.6 years (interquartile range [IQR], 30.6 to 41.6 years). The median CD4 cell count was 224 cells/µL (IQR, 107.0 to 360.5 cells/µL) (with a wide range: 4.0 to 824.0 cells/µL), which was similar in both groups. The median time since being diagnosed with HIV infection was 12.0 months (IQR, 6.0 to 53.5 months; range, 1.0 to 204.0 months), and the median duration of KS lesions before diagnosis was 4.8 weeks (IQR, 3.7 to 8.8 weeks; range, 2.0 to 157.9 weeks). The saturation of arterial oxygen, as measured by a pulse oximeter, and creatinine levels were normal throughout the study.

**Table 1:**
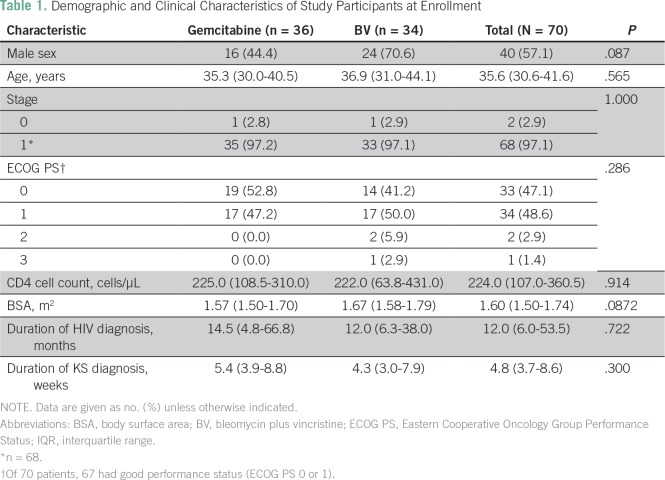
Demographic and Clinical Characteristics of Study Participants at Enrollment

### Response Rates

There was no statistical difference between response rates to treatment in both arms (Table 2). The response rate (CR plus PR) was 86.1% for gemcitabine and 76,4% for BV. Four participants receiving gemcitabine and seven participants receiving BV remained stable; one participant from each arm had progressive disease.

**Table 2:**
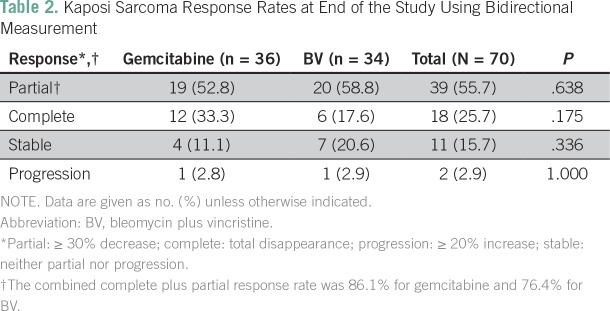
Kaposi Sarcoma Response Rates at End of the Study Using Bidirectional Measurement

### Adverse Events

Hematologic and peripheral neuropathy effects assessed by Common Terminology Criteria for Adverse Events version 4 showed no evidence of differences in adverse events (AEs) indicated by laboratory studies, namely, anemia, and neutropenia (Table 3). No participant died while receiving treatment during the study.

**Table 3:**
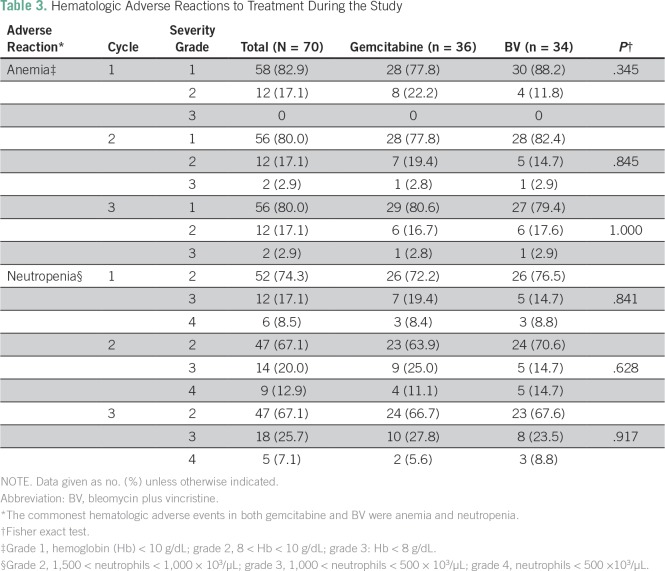
Hematologic Adverse Reactions to Treatment During the Study

Occurrence of peripheral neuropathy was not significantly different between the two study arms (*P* = .503; Table 4). Grades 3 and 4 neuropathy developed after cycle 3 of chemotherapy.

**Table 4:**
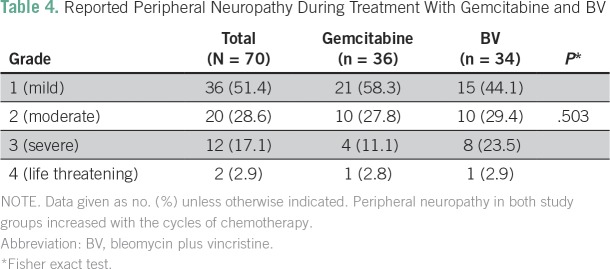
Reported Peripheral Neuropathy During Treatment With Gemcitabine and BV

### Health-Related QoL

**Fig 2:**
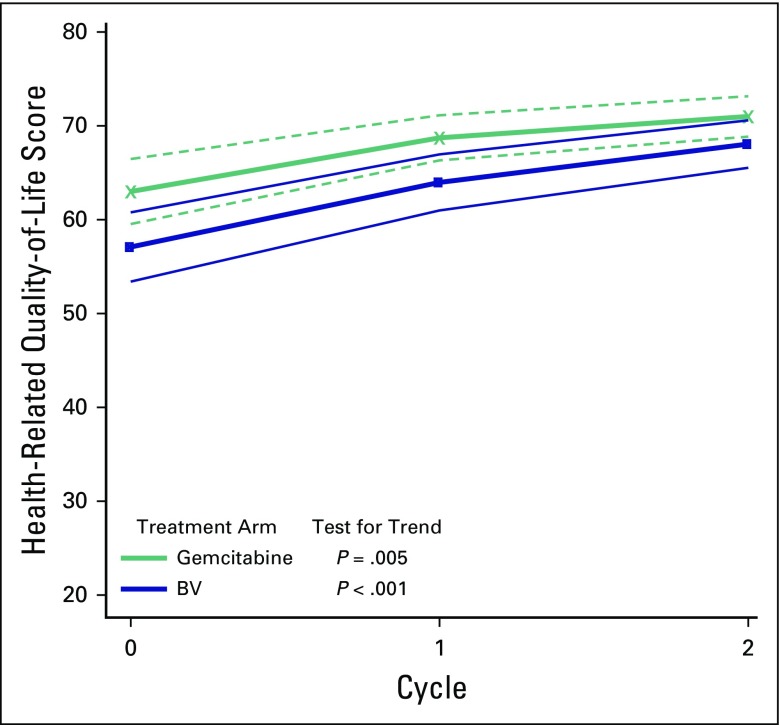
Trajectories of health-related quality-of-life scores using the Functional Assessment Cancer Therapy-Kaposi Sarcoma score with the corresponding 95% CIs over follow-up. Participants in the bleomycin plus vincristine (BV) and gemcitabine arms improved in function, but neither arm was statistically improved compared with the other, because the CIs overlap. Thin black lines represent BV CIs; dashed red lines represent gemcitabine CIs.

Study participants responded to every question on the FACT-KS questionnaire in the four domains of QoL after each cycle of chemotherapy but before the next cycle. Participants in the gemcitabine arm gained 8.0 points and those in the BV arm gained 11.0 points after three cycles of chemotherapy (*P* = .009 and .001, respectively). The trajectories in Figure 2 show an increasing trend in the health-related QoL scores for both study arms.

As shown in Table 5, participants in both study arms reported significant improvement in physical well-being during cycles 1 and 2 (*P* < .05). Similarly, significant improvement was noted in functional well-being during cycles 1 and 2. After cycle 3, there was no significant improvement in these two domains. Significant improvement did not occur in the other two domains (ie, social and family well-being, and emotional well-being) in all the treatment cycles (*P* > .05).

**Table 5:**
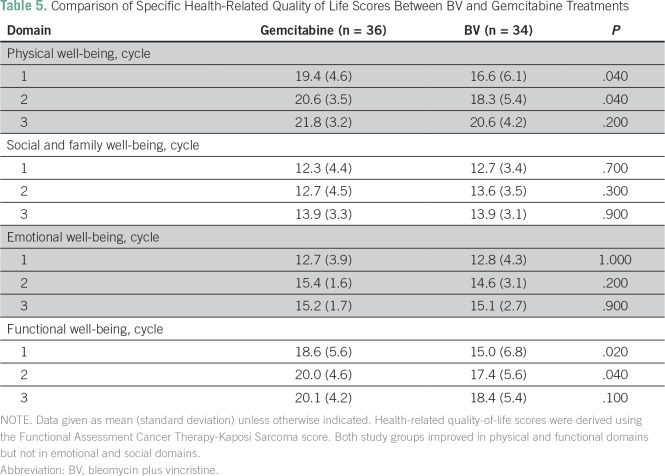
Comparison of Specific Health-Related Quality of Life Scores Between BV and Gemcitabine Treatments

## DISCUSSION

In this prospective phase IIA study evaluating modified-dose gemcitabine therapy, we found clinically objective response with an overall response rate of 86%. In addition, we noted a 76.4% overall response rate for BV. Similar studies in Malawi reported total response rates of 64% to vincristine and 74% to BV,^10,12^ whereas a related study conducted in the United States reported a response rate of 72%.^11^ Evaluation for response at 6 weeks is adequate for a phase IIA study, which usually involves few participants followed for a short time. This is also appropriate for AIDS-KS, for which chemotherapy is adjuvant and long-term treatment is cART.

In our trial, the frequency and types of AEs were similar between the gemcitabine and BV arms. None of these AEs were severe or life threatening. Reported AEs involved the nervous and hematologic systems but were mild in both treatment arms. As expected, more severe neurotoxicity was reported in the BV arm (23.5% *v* 11.1%; however, overall neurotoxicity was similar in both arms (*P* = .503). This has been previously reported for vincristine.^25^ None of the participants developed clinical pulmonary toxicity, which is typically not seen at such a low dosage of bleomycin.^26^ According to the gemcitabine package insert, hematologic AEs occur in 89% of users, whereas neurologic AEs occur in 35%^27^; in this study, 58% of AEs were hematologic and 86% were neurologic. The lower incidence of hematologic toxicity is related to the biweekly administration of gemcitabine instead of weekly regimen. As a whole, the study population seemed to have greater-than-anticipated chemotherapy-related neurologic events, perhaps because of prior therapy with cART.

When using the FACT-KS questionnaire, a clinically meaningful change is defined as an increase by 5 points.^23^ As previously stated, participants on gemcitabine and BV gained 10 and 13 points, respectively, after three cycles of chemotherapy (*P* = .0009 and .001, respectively). The domains that contributed to improved QoL scores were physical well-being and functional well-being. There was no change in the other two domains. The study participants appeared to prioritize physical and functional well-being, perhaps because most of them were self-employed. This is an area that requires additional research. QoL responses have been criticized for being subjective in cancer studies because patients overestimate benefit from treatment that causes under-reporting of negative changes. In this study, this limitation was mitigated by improvement in Eastern Cooperative Oncology Group performance status scores after chemotherapy.

This study reports results of a randomized trial of gemcitabine as first-line therapy for AIDS-KS. Like similar phase IIA studies, we had limited follow-up, with analysis performed after three cycles of therapy.

In conclusion, gemcitabine has demonstrated activity in cART-resistant AIDS-KS in a population in sub-Saharan Africa. Additional studies on gemcitabine to treat AIDS-KS are recommended.
